# Old Issues and New Perspectives on Endometrial Cancer Therapy: How Molecular Characteristics Are Changing the Therapeutic Pathway

**DOI:** 10.3390/cancers16101866

**Published:** 2024-05-14

**Authors:** Daniela Luvero, Gianna Barbara Cundari, Fernando Ficarola, Francesco Plotti, Corrado Terranova, Roberto Montera, Giorgio Bogani, Adele Silvagni, Federica Celoro, Roberto Angioli

**Affiliations:** 1 Department of Gynaecology, Fondazione Policlinico Universitario Campus Bio-Medico, Via Alvaro del Portillo 200, 00128 Roma, Italy; 2 Research Unit of Gynaecology, Department of Medicine and Surgery, Università Campus Bio-Medico Di Roma, Via Alvaro del Portillo 21, 00128 Rome, Italy; 3 Unit of Gynaecology, Department of Surgical and Medical Sciences and Translational Medicine, Sant’Andrea Hospital, Sapienza University of Rome, 00189 Rome, Italy; 4 Gynecological Oncology Unit, Fondazione IRCCS Istituto Nazionale dei Tumori di Milano, 20133 Milano, Italy

**Keywords:** endometrial neoplasms, immunotherapy, immune checkpoint inhibitors, antineoplastic agents, microsatellite instability

## Abstract

**Simple Summary:**

Endometrial cancer is the sixth most common cancer among women worldwide, with an increasing mortality rate. The standard treatment of patients with advanced or recurrent endometrial cancer has not changed significantly in recent decades. However, in recent years, immunotherapy has emerged as a promising strategy that harnesses the body’s immune system against cancer, particularly in advanced or recurrent cases, offering patients the possibility of better results and longer survival. Our critical review analyses recent findings in the literature in this field, focusing on the role of immunotherapy in the treatment of this disease. In particular, it focuses on the importance of molecular biology and highlights how this can change the choice of treatment, providing insights for future therapeutic strategies.

**Abstract:**

The Cancer Genome Atlas (TCGA) has radically changed the history of endometrial cancer by outlining a new classification, based on its molecular characteristics. In the field of oncology, we are approaching the new era of molecular biology, particularly regarding endometrial cancer, with the increasing importance of targeted therapy. This paper is a review of phase III randomized controlled trials published in English between January 2019 and December 2023, comparing drugs of interest with standard adjuvant treatment and molecular subtypes in endometrial cancer. The use of immunotherapy alone or in combination with chemotherapy as therapy in patients with recurrent or advanced primary or metastatic endometrial cancer significantly improves the prognosis of these patients. The results show greater efficacy of all proposed treatments for mismatch repair deficiency (dMMR/MSI-H) patients compared to mismatch repair proficiency (pMMR) patients. Progression-free survival (PFS) and overall survival (OS) are better in dMMR patients in all studies analysed. Immunotherapy has the potential to revolutionize the gynaecological cancer treatment landscape, offering a new pathway and new hope for endometrial cancer patients, improving their outcomes in the future. Given the exciting results obtained in dMMR/MSI-H patients, MMR status should be investigated in every patient with advanced endometrial cancer at the time of diagnosis.

## 1. Introduction

Endometrial cancer is the sixth most common cancer among women worldwide with an incidence of about 400,000 new cases in 2020 [1]. In Europe, endometrial cancer is the leading gynaecological cancer by incidence with an age-standardized rate of 14.3 per 100,000 in 2022 [2]. It is a cancer whose incidence and mortality are increasing worldwide; in particular, mortality rates continue to increase by about 1% per year [3,4]. International organizations (WHO, FIGO, and ESGO) have redefined the classification of endometrial cancer, focusing on new molecular targets and establishing new risk classes to identify patients fit for adjuvant treatment. In fact, the classification of endometrial cancer has undergone significant changes, moving from a purely histopathological to a molecular-based risk stratification.

In 1983, the first classification was proposed by Bokhman’s differentiated endometrial cancer into type I and type II based on the expression of oestrogen receptors [5]. In 2014, the WHO classification defined a risk stratification based on histopathological features with different important prognoses [6]. The Cancer Genome Atlas (TCGA) radically changed the history of this cancer, outlining a new classification of endometrial cancer based on its molecular features [7]. This classification identified four subtypes of endometrial cancer: POLE (DNA polymerase epsilon) mutated, microsatellite instability high (MSI-H), copy number low, and copy number high [7]. 

Based on their molecular characteristics and different prognostic patterns, these tumours have been divided into “hot tumours” and “cold tumours” [8]. POLE mutated and microsatellite instability high (MSI-H) are defined as “hot tumours” as they are full of inflammatory infiltrate, so they present characteristics compatible with adaptive immune resistance. These tumours respond well to immunotherapy, directed against specific surface antigens such as PD-1 and PDL-1. Copy number low and copy number high are termed “cold tumours” as they are poor in lymphocyte infiltrate and therefore benefit more from combination therapies including radiotherapy. 

With the 5th edition of the *WHO Classification of Female Genital Tumours* published in 2020, attention was drawn to the central role of molecular genetics in the classification of endometrial cancer and integration of molecular-histologic typing in endometrial carcinoma, which was recommended for high-grade endometrial carcinoma [9]. The algorithm recommends using POLE mutation analysis followed by immunohistochemistry for mismatch repair protein assessment and finally p53 to stratify tumours into four groups with different prognoses [9]. 

A big step forward has been made recently, in 2023, with the publication of the new FIGO guidelines, which integrate the new histopathological stage, tumour patterns, and molecular classification [10]. According to this recent FIGO classification, when feasible, the addition of the molecular subtype to staging criteria allows for a better prognosis prediction of the disease. When molecular classification reveals POLE mutation (POLEmut) or p53 abnormal (p53abn) status, the FIGO stage is modified in early-stage disease. Mismatch repair deficiency (dMMR) or no specific molecular profile (NSMP) status does not alter early FIGO stages; however, these molecular classifications should be recorded for data collection purposes. FIGO stages III and IV are based on surgical/anatomical findings. Stage category is not altered by molecular classification, but the latter should be recorded if known [10]. With the new classification, the extent of myometrial invasion is consistently recognized as an essential prognostic risk factor, to be expressed as the percentage of the overall myometrial thickness infiltrated by carcinoma, using three categories: none; <50%; or ≥50% [11,12,13,14,15]. 

The goal of the current modifications to the endometrial staging system is improving the definition of prognostic groups and creating substages that could appear more appropriate for surgical, radiation, and systemic therapies [16]. In the oncology field, we are approaching the new era of molecular biology, particularly regarding endometrial cancer, increasing the importance of targeted therapy. Indeed, the therapeutic choice is increasingly being directed towards a subtype-specific approach. In this scenario, the introduction of immunotherapy, in combination with chemotherapy or in monotherapy, represents a promising new field of interest in endometrial cancer research. 

The aim of this review is to analyse new discoveries in the field of endometrial cancer therapy, focusing on recent scientific trials and delving into how molecular biology is redefining the meaning of “target-therapy” and changing the therapeutic pathway for patients.

## 2. Materials and Methods

This review was carried out following the standard medical database, MEDLINE (Platform: PubMed). We searched the terms [“endometrial neoplasms” OR “endometrial cancer” OR “uterine cancer”] AND [“immune checkpoint inhibitors” OR “ICIs” OR “immunotherapy” OR “molecular subtyping”] for articles published in English between January 2019 and December 2023. All phase III randomized controlled trials comparing the medications of interest with standard adjuvant treatment and molecular subtypes in endometrial cancer were included. Phase I–II trials, meta-analyses, reviews, case reports, correspondences, personal opinions, and in vitro/animal studies were excluded. Ongoing trials, related to emerging medical therapy in endometrial cancer, were explored on Clinicaltrials.gov (accessed on 18 April 2024). Ongoing trials, although the first partial results were published, were excluded from the analysis but considered in the discussion. For the selected studies, the following data were collected: trial name, first author, year of publication, number of treated patients, target population, administered drugs and dosage, and primary and secondary endpoints. Specifically, progression free survival (PFS), overall survival (OS), discontinuation rate, dose reduction, and temporary interruption rates were sought in each study. An analysis of the main toxicities was performed. For each study, results were analysed in the different mismatch repair-deficient (dMMR) and mismatch repair-proficient (pMMR) populations, when applicable. Two reviewers (D.L. and G.B.C.) working independently identified eligible studies by screening titles and abstracts. If a study was deemed relevant, the manuscript was obtained and reviewed in full text. All authors independently assessed the quality of the studies, taking into consideration representativeness and quality of data. Two reviewers (D.L. and G.B.C.) independently assessed the risk of bias. In case of disagreement, a third reviewer (F.F.) was consulted.

## 3. Results

**Figure 1:**
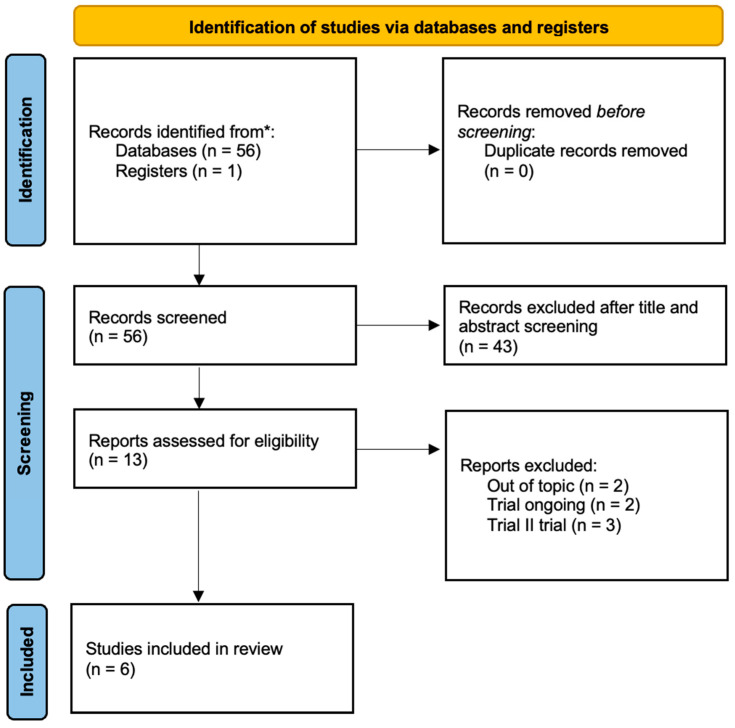
Flowchart for study selection of the review. * Platform: PubMed.

From our search, 56 studies were identified from 2019 to 2023. After title/abstract screening, 13 eligible articles were selected. According to inclusion and exclusion criteria, two articles were removed because they were out of topic, three articles because they were phase II studies, and two articles because the trials were ongoing. At the end of the screening, a total of six randomized phase III studies were included in our review (Figure 1) [17,18,19,20,21,22].

Anti-PD1 agents were analysed in five studies, in particular Dostarlimab in one study [17] and Pembrolizumab in four studies [18,20,21,22]. In three studies, Pembrolizumab was analysed in combination with the tyrosine kinase inhibitor (TKI) Lenvatinib [20,21,22]. The anti-PD-L1 agent represented by Durvalumab was analysed in one study, also in combination with the PARP-inhibitor Olabarip [19]. No single-agent anti-CTLA-4 study was found. 

Dostarlimab was started at a dosage of 500 mg q3w and then continued at 1000 mg every 6 weeks (q6w) for 3 years [17]. Pembrolizumab was administered every 3 weeks (q3w) at the fixed dosage of 200 mg in four studies [18,20,21,22]. In the study published by Eskander RN et al., this dosage of Pembrolizumab was administered for 6 cycles followed by 400 mg every 6 weeks (q6w) for 14 cycles [18]. In three studies, patients received up to 35 doses of Pembrolizumab in combination with Lenvatinib administered at a dosage of 20 mg orally once daily [20,21,22]. Durvalumab was administered at a fixed dosage of 1120 mg q3w for six cycles followed by a dosage of 1500 mg q4w alone or in combination with Olaparib 300 mg orally twice daily [13]. The two studies published by Makker V. et al. and Yonemori K et al. in 2022 and 2023 [21,22] represented the secondary analysis of data about efficacy and safety from study 309/KEYNOTE-775. Yonemori [22] showed the subgroup analysis of the Japanese population included in study 309/KEYNOTE-775 [20]. 

In these six phase III trials included in our analysis, a total of 2855 patients, ranging from 104 to 827, were treated. Progression-free survival (PFS)—defined as the time from randomization to disease progression or death, whichever occurred first—was the primary endpoint in all studies [17,18,19,20,21,22]. Overall survival (OS), defined as the time from randomization to death, was assessed as a co-primary endpoint with PFS in four studies [17,20,21,22] and as a secondary endpoint in the other two studies [18,19]. Safety was examined as a secondary endpoint in all six studies analysed [17,18,19,20,21,22]. Overall response rate (ORR) and quality of life were most investigated among the secondary endpoints; in particular, quality of life was considered in five studies [17,18,19,20,21]. Analysis of tumour molecular characteristics was performed in all studies. Mismatch repair (MMR) and microsatellite status was performed in all patients. Specifically, 616 mismatch repair-deficient (dMMR) patients were analysed. Table 1 shows the main characteristics of the included studies.

In each study, a primary and secondary endpoint analysis was performed for the dMMR population subcategory. In fact, Mirza et al. in the RUBY trial reported the Kaplan–Meier estimated probability of progression-free survival at 24 months as 36.1% in the overall population and 61.4% in the dMMR population and estimated the probability of overall survival at 24 months as 71.3% in the overall population and 83.3% in the dMMR population [17]. Eskander et al. in the NRG-GY018 trial and Westim et al. in the DUO-E/ENGOT-EN10 trial reported the Kaplan–Meier estimated probability of progression-free survival at 12 months represented in the dMMR population as 74% versus 67.9% (Durvalumab group) versus 70% (Durvalumab + Olaparib) [18,19]. Three studies reported median PFS ranging from 5.6 to 6.7 months in the pMMR population [20,21,22]. Westim et al. in the DUO-E/ENGOT-EN10 trial reported the Kaplan–Meier estimated probability of overall survival at 12 months represented as 84.2% (Durvalumab group) and 87.7% (Durvalumab + Olaparib) [19]. Three studies reported a median OS ranging from 16.7 to 18.0 months in the pMMR population [20,21,22]. In all studies, discontinuation rates ranged from 17.4% to 57%. Dose reduction ranged from 6% to 82.7%. Temporary discontinuation ranged from 54.5% to 71.9%. Table 2 shows the main results of the included studies. 

Regarding the safety, in the studies analysed, from 93.5% to 100% of patients developed treatment-related adverse effects (TRAEs), of which 54.9% to 90.4% were ≥G3. Adverse effects leading to death ranged from 0% to 6.4%. Fatigue was one of the most common adverse effects, ranging from 43% to 71.6%. Table 3 shows an analysis of treatment-related adverse effects (TRAEs).

A sub-analysis of haematological toxicities shows that anaemia and leukopenia represent the types of toxicity that most caused adverse events ≥ G3 in both groups of the RUBY trial: anaemia occurs in 37.8% of patients in the Dostarlimab group versus 42.3% in the placebo group [17]. In the NRG-GY018 trial of Eskander et al., anaemia was one of the most common toxicities, occurring in 57.8% of the Pembrolizumab dMMR group and 55.1% of the Pembrolizumab pMMR group [18]. However, the percentages were comparable to the placebo group. The second type of haematological toxicity developed by the patients in this study was thrombocytopenia with higher rates than in the placebo group: 34.9% in the Pembrolizumab dMMR group versus 29.2% in the placebo group; and 30.1% in the Pembrolizumab pMMR group versus 21.5% in the placebo group [18]. In the DUO-E/ENGOT-EN10 trial of Westim et al., anaemia represented the most common toxicity developed by patients, especially in the Durvalumab + Olaparib group (61.8%) [19]. Patients also developed neutropenia: 41.6% in the Durvalumab + Olaparib group, comparable to the control group (41.5%) but slightly higher than in the Durvalumab group (35.7%). Thrombocytopenia occurred in 29.8% of the Durvalumab + Olaparib group, comparable to the Durvalumab group (28.1%) but higher than the control group (22%) [19]. Contrarily, in the 309-KEYNOTE-775 trial of Makker et al., including the secondary analysis and the Japanese subset analysis, haematological toxicities were not among the most common [20,21,22]. Anaemia was observed in 26.1% of patients in the Lenvatinib plus Pembrolizumab group, a much lower percentage than in the chemotherapy group where anaemia was observed in 48.7%. Neutropenia also showed low rates in the Lenvatinib plus Pembrolizumab group (7.4%) compared to the chemotherapy group (33.8%) [20,21,22].

A sub-analysis of extra-haematological toxicities shows that two of the most common immune-related adverse events are hypothyroidism and myalgia. As for hypothyroidism, in the Ruby trial it occurred in 11.2% of the patients in the Dostarlimab group [17]. In the NRG-GY018 trial, hypothyroidism occurred in 12.8% of the patients in the Pembrolizumab dMMR group and 13.4% in the Pembrolizumab pMMR group [18]. The incidence of hypothyroidism was very high in the 309-KEYNOTE-775 trial of Makker et al.: 57.6% (grade 1 in 17.2% and grade 2 in 38.9%) among patients who received Lenvatinib plus Pembrolizumab [20]. Regarding myalgia, in the Ruby trial it occurred in 26.1% of the patients in the Dostarlimab group [17]. A similar percentage (26.6%) of patients developed myalgia in the Pembrolizumab dMMR group in the NRG-GY018 trial, whereas a slightly lower percentage of myalgia (16.3%) was observed in the pMMR group [18]. In the 309-KEYNOTE-775 trial of Makker et al., in which Pembrolizumab was used as in the NRG-GY018 trial, myalgia was not mentioned among the most common adverse effects [20]. Among the rarer immune-related adverse events, hypophysitis was mentioned in the NRG-GY018 trial of Eskander et al., which occurred in 0.7% of the Pembrolizumab pMMR group (all of G ≥ 3) and in none of the patients in the Pembrolizumab dMMR group [20].

## 4. Discussion

Endometrial cancer is a disease that radically changes the well-being of women who are afflicted by it. Advanced, recurrent, or metastatic forms of endometrial cancer are largely impacted both in terms of morbidity and mortality and in terms of quality of life. 

In this scenario, the new therapeutic strategies proposed for the treatment of these patients may change the history of this pathology. As shown by the results of our review, the use of immunotherapy alone or in combination with chemotherapy as therapy in patients with recurrent or in primary advanced or metastatic endometrial cancer significantly improves the prognosis of these patients. Historically, chemotherapy alone has had limited efficacy in patients with endometrial cancer with a survival in patients with endometrial cancer of approximately 12 months [23,24,25]. The results of the review also emphasize the importance of patient stratification, based on the molecular characteristics of the endometrial tumour, which allows for targeting the choice of therapy and defining a target therapy tailored to the patient’s characteristics. All studies included in the review stratified patients based on molecular characteristics, differentiating dMMR patients from pMMR patients. An analysis of the efficacy of the proposed treatments was carried out in each of these subcategories. The results show a higher efficacy of all proposed treatments for dMMR patients than for pMMR patients. PFS and OS are better in dMMR patients in all studies analysed. The PFS and OS of the analysed studies are not fully comparable, considering the heterogeneity of the sample, the study design, and the different follow-ups considered. It is possible to compare the results of the NRG-GY018 trial and the DUO-E/ENGOT-EN10 trial, which both show a follow-up of 12 months [18,19]. In dMMR patients, the PFS rate is 74% (Pembrolizumab group) in the NRG-GY018 trial and 70% (Durvalumab + Olaparib group) versus 67.9% (Durvalumab group) in the DUO-E/ENGOT-EN10 trial [18,19]. In the RUBY trial of Mirza et al., the drug was significantly more effective in the dMMR population than in the overall population with a PFS rate of 61.4% versus 36.1% at 24 months and an OS rate of 83.3% versus 71.3% at 24 months [17]. The 309-KEYNOTE-775 trial of Makker et al., the secondary analysis, and the Japanese subset analysis analysed the differences in terms of PFS and OS between the subgroup of pMMR patients and the overall population, confirming reduced efficacy in pMMR patients compared to the overall population [20,21,22]. 

The discontinuation rate was comparable in all studies included in the review: the highest rate was observed with Pembrolizumab (57%) in the NRG-GY018 trial and the lowest rate was observed with Dostarlimab (17.4%) in the RUBY trial [17,18]. As shown in the RUBY trial, Dostarlimab was therefore the best tolerated drug among those analysed that resulted in a lower discontinuation rate [17]. It is interesting to analyse that in the secondary analysis of the 309-KEYNOTE-775 trial, there were very high rates of dose reduction and temporary discontinuation (72.2% and 71.9%, respectively) [21]. As shown in the 309–KEYNOTE-775 trial, Pembrolizumab in combination with Lenvatinib shows poor tolerability compared to the other drugs analysed [20]. This is confirmed by the analysis of grade ≥3 toxicities. In all studies analysed, more than 50% of the patients showed grade ≥ 3 toxicity. The best tolerated drug was Durvalumab from the DUO-E/ENGOT-EN10 trial with a grade ≥ 3 toxicity rate of 54.9% [19]. The combination of Pembrolizumab and Lenvatinib from the 309–KEYNOTE-775 trial had a poorer tolerability with a grade ≥3 toxicity rate of 90.1%, 90.4% in the Japanese subset, and an adverse effect causing death rate of 6.4% [22]. With the use of the above-mentioned drugs, attention must always be paid to the management of the patient, considering the high rate of toxicities, even of grade ≥3, that can occur with the use of immunotherapy. As highlighted in the results of our review, immune-related toxicities, such as hypophysitis, can also occur, and although these are rare, they must always be looked for in relation to the patient’s clinical aspects and symptoms. 

It is important to highlight that even though the period chosen for the selection of clinical trials for inclusion in the review runs from 2019 to 2023, the studies meeting the inclusion criteria were all published in 2022 and 2023. This shows how clinical trials in this field are constantly increasing and how the focus of clinical research for endometrial cancer is increasingly shifting towards molecular genetics. Indeed, the last two years are those in which the literature production in this field has intensified the most. Moreover, none of the studies mentioned has made a more in-depth search for the molecular characteristics of patients: the studies are limited to differentiating dMMR patients from pMMR patients. It would be interesting to observe how the efficacy of the individual therapies analysed changes depending on the specific molecular subcategory the patient belongs to, thus also looking for polymerase ε (POLE) and p53 mutations.

### 4.1. Strengths and Limitations

All therapies analysed demonstrate efficacy in treating advanced or recurrent endometrial carcinoma compared to standard chemotherapy, especially in patients with dMMR, highlighting the essential role of molecular diagnostics integration in patient therapeutic decision-making. The strengths of our review lie in the utilization of only randomized phase 3 clinical trials, thus holding implications for clinical practice, the inclusion of recent studies, and the incorporation of studies utilizing the molecular classification of endometrial tumours, emphasizing its importance in outcomes. However, our review presents several limitations, including a limited number of included studies and their heterogeneity. Additionally, some data are incomplete and not entirely comparable; a longer follow-up period is necessary to better elucidate the role of certain drugs in the prognosis of these patients.

### 4.2. Future Perspectives

The new FIGO endometrial cancer classification also focuses on the molecular characteristics of the tumour, redefining the risk classes also depending on the molecular histotype [10]. However, a direct correlation with therapeutic strategies depending on the molecular histotype of endometrial cancer is lacking in the literature. Several ongoing trials are precisely analysing this aspect. Among them is the DOMENICA study (GINECO-EN105b/ENGOT-en13 study), the recruitment of which started in April 2022 [26]. This compares Dostarlimab versus chemotherapy alone in first-line advanced/metastatic in MMR-deficient (MMRd) endometrial cancer (EC) patients, focusing on a specific patient category. 

The RAINBO study is one of the most promising ongoing trials [27]. This program examines four adjuvant treatment strategies targeting different molecular classes of endometrial cancer following surgical resection. Specifically, the RAINBO program comprises four international clinical trials:

- The randomized phase III p53abn-RED trial for women with invasive stage I–III p53abn endometrial carcinoma compares adjuvant chemoradiation followed by Olaparib for 2 years with adjuvant chemoradiation alone.
- The randomized phase III MMRd-GREEN trial for women with stage II (with lymphovascular space invasion (LVSI)) or stage III endometrial carcinoma with mismatch repair deficiency (dMMR) compares adjuvant radiation therapy with concurrent and adjuvant Durvalumab for 1 year with radiation therapy alone.
- The randomized phase III NSMP-ORANGE trial is a treatment de-escalation study for women with oestrogen receptor-positive endometrial carcinoma in stage II (with LVSI) or stage III without a specific molecular profile (NSMP) comparing radiation therapy followed by progestin for 2 years with adjuvant chemoradiation therapy.
- The POLEmut-BLUE trial is a phase II study investigating the safety of adjuvant therapy de-escalation for women with POLEmut endometrial carcinoma in stage I-III: no adjuvant therapy for low-risk disease and no adjuvant therapy or radiation therapy alone for higher-risk disease.

The overarching RAINBO program will combine data from all participants to conduct translational research and evaluate adjuvant therapy based on molecular classes in terms of efficacy, toxicity, quality of life, and cost-effectiveness. Results are expected in 2028 and could potentially revolutionize the treatment landscape for this tumour [27].

The PORTEC-4a trial is an ongoing study that analyses different adjuvant therapeutic strategies after surgery for endometrial cancer, based on the tumour’s molecular characteristics [28]. Specifically, PORTEC-4a is the first randomized trial to investigate the use of a molecular-integrated risk profile to determine adjuvant treatment in endometrial cancer. The study compares rates of vaginal recurrence in women with high-intermediate risk endometrial carcinoma, treated after surgery with recommendations based on the molecular risk profile for observation, vaginal brachytherapy, or external pelvic radiotherapy versus standard adjuvant vaginal brachytherapy. The goal is to spare many patients the morbidity of adjuvant treatment and reduce healthcare costs while maintaining the same local disease control and recurrence-free survival [28].

The PROBEAT study is a phase III randomized trial evaluating personalized adjuvant treatment based on the WHO-approved molecular classification in Chinese patients with endometrial cancer [29]. It investigates adjuvant treatment in patients with high-intermediate risk (HIR), intermediate risk (IR), or low-intermediate risk (IR) endometrial cancer. The study plans to classify all tumour tissues into four molecular subtypes (POLEmut, MMRd, p53abn, or NSMP). Patients will be randomly assigned in a 2:1 ratio to one of the two experimental arms and will receive adjuvant treatment based on the molecular profile (observation in the POLEmut subgroup, vaginal brachytherapy in the MMRd or NSMP subgroups, or chemoradiotherapy in the p53abn subgroup) or to the standard arm and receive standard adjuvant radiotherapy [29].

A therapeutic strategy that seems to be promising in the treatment of primary advanced or recurrent EC is to combine immunotherapy with a PARP inhibitor, as demonstrated by the recent presentation of the first interim results of RUBY Part 2 [30]. This study shows significant and clinically meaningful improvement in PFS for Dostarlimab + chemotherapy followed by Dostarlimab + niraparib in the overall and MMRp/MSS populations. The trial is ongoing for OS follow-up. The safety profile observed was generally consistent with the known safety profiles of the individual agents. So, these outcomes demonstrate a potential role for PARP inhibitor maintenance in patients receiving Dostarlimab plus chemotherapy, in particular for MMRp/MSS disease.

Among the future therapeutic prospects for endometrial tumours, as knowledge of molecular characterization increases, additional targets with potential therapeutic implications for specific subgroups of endometrial cancer are being identified [16]. The overlap between the various molecular pathways involved in endometrial cancer makes it possible to combine new agents to develop effective therapies. Recent phase 1–2 studies have examined the efficacy of molecularly targeted therapies. Among these, inhibitors of the PI3K/AKT/mTOR pathway have been one of the main agents widely studied. Particularly promising results have been obtained from the VICTORIA study [31]. Based on the premise that the dysregulation of the PI3K/AKT/mTOR pathway observed in endometrial carcinoma determines hormonal resistance, the combination of mTOR inhibitor with endocrine therapy has been utilized. This study demonstrated that adding vistusertib (mTOR inhibitor) to anastrozole improved the progression-free rate at 8 weeks (8wk-PFR), overall response rate, and PFS for patients with endometrial cancer and had manageable adverse events [31]. Another recent phase 2 study analysed the efficacy of sapanisertib (selective dual inhibitor of mTORC1/2) alone, or in combination with paclitaxel or TAK-117 (a selective small molecule inhibitor of PI3K), versus paclitaxel alone in advanced, recurrent, or persistent endometrial cancer [32]. This study supports the inclusion of chemotherapy combinations with investigational agents for advanced or metastatic disease, showing manageable toxicity. However, not all phase 1–2 studies have demonstrated proven efficacy of molecularly targeted therapies. In particular, the combination of monalizumab (anti-NKG2A/CD94) and Durvalumab (anti-programmed death ligand-1) has shown modest efficacy in the treatment of solid tumours [33]. Similarly, avelumab combined with utomilumab (a 4-1BB agonist), PF-04518600 (an OX40 agonist), and radiotherapy in patients with recurrent gynaecologic malignancies did not produce a significant treatment response [34].

Another widely studied therapeutic target for endometrial cancer is human epidermal growth factor receptor 2 (HER2), a receptor tyrosine-protein kinase encoded by ERBB2. Trastuzumab, a humanized monoclonal antibody targeting Her2/Neu, has been studied in advanced/recurrent uterine-serous carcinomas in combination with carboplatin and paclitaxel in a phase II study that showed clinical benefit [35].

## 5. Conclusions

The standard treatment of patients with advanced or recurrent endometrial cancer has not significantly changed over the past few decades. The new FIGO guidelines, which integrate the new histopathological stage, tumour patterns, and molecular classification, are radically changing this scenario and the development of novel therapies tailored to individual tumour characteristics [10].

The primary treatment is surgical, involving total hysterectomy and salpingo-oophorectomy, currently often with the detection of sentinel lymph nodes, possibly with radiation and chemotherapy. The tumour microenvironment plays a significant role in endometrial cancer progression, involving immune cells and stromal cells [36].

In recent years, immunotherapy has emerged as a promising strategy capable of stimulating the body’s immune system against cancer, particularly in advanced or recurrent cases, offering patients the chance to achieve better outcomes and prolong survival. Immunotherapy proves particularly effective in tumours with specific molecular characteristics. Indeed, immune checkpoint inhibitors as single agents have demonstrated higher responses among dMMR/MSI patients. Conversely, pMMR/MSS patients derive greater benefits from the combination of immunotherapy and chemotherapy, despite the toxicity being worse compared to immunotherapy alone. Close clinical monitoring and careful toxicity management are therefore fundamental. These studies have led to the approval of Dostarlimab and Pembrolizumab in routine clinical practice for patients with advanced/recurrent pre-treated endometrial carcinoma [17,18].

In conclusion, immunotherapy has the potential to revolutionize the landscape of gynaecological cancer treatment, offering a new pathway and hope for patients with endometrial cancer, improving their outcomes in the future. Equally crucial is the identification of other reliable predictive biomarkers, in addition to MMR status, to select patients who are most likely to benefit from immunotherapy, as well as better understanding the mechanisms of resistance that some patients develop during treatment. For these reasons, further studies are needed to identify, especially in patients with unfavourable prognosis (pMMR), innovative strategies to improve treatment outcomes. Considering the phase 1–2 studies examining the role of molecular-targeted therapy in endometrial cancer present in the literature, further studies are necessary to investigate the efficacy of new molecules, and especially phase 3 studies are needed to ascertain their effectiveness and applicability in clinical practice.

In the future, we think that combining immunotherapies with traditional treatments like surgery, chemotherapy, and radiation therapy may enhance overall treatment efficacy and really change the patient’s pathway already from the first line of treatment.

## Figures and Tables

**Table 1:** Characteristics of studies that compared immunotherapy with standard adjuvant treatment and molecular subtypes in endometrial carcinoma.

Authors	Study Name	Patients	Target Population	Administered Drug	Primary EP	Secondary EP
Mirza et al., 2023 [17]	RUBY, NXT03981796	N = 494 Dostarlimab: 241/494 dMMR: 118/494	Primary advanced or recurrent EC [FIGO stage III or IV]	Dostarlimab 500 mg or placebo + PBC q3w × 6c -> Dostarlimab 1000 mg or placebo q6w × 3 y	PFS, OS	ORR, Safety, DC, RD, TSPD, QoL
Eskander et al., 2023 [18]	NRG-GY018, NCT03914612	N = 816 Pembrolizumab: 405 dMMR: 225	Advanced, metastatic, or recurrent EC (except carcinosarcoma) [FIGO stage III or IV]	Pembrolizumab 200 mg or placebo + PBC q3w × 6c -> Pembrolizumab 400 mg or placebo q6w × 14c	PFS	OS, Safety, QoL
Westim et al., 2023 [19]	DUO-E/GOG-3041/ENGOT-EN10 trial/NCT04269200	N = 718 Durvalumab: 238/718 Durvalumab + Olaparib: 239/718 dMMR:143/718	Advanced or recurrent EC (except sarcoma) [FIGO stage III or IV]	PBC + placebo -> placebo; PBC + Durvalumab 1120 mg q3w × 6c -> Durvalumab 1500 mg q4w + placebo; PBC + Durvalumab 1120 mg q3w × 6c -> Durvalumab 1500 mg q4w + Olaparib 300 mg/twice daily	PFS	OS, Safety, QoL
Makker et al., 2022 [20]	309–KEYNOTE-775, NCT03517449	N = 827 Lenvatinib + Pembrolizumab: 411/827 dMMR: 130/827	Advanced, recurrent, or metastatic endometrial cancer (except carcinosarcoma and sarcoma)	Lenvatinib 20 mg once daily + Pembrolizumab 200 mg q3w or TPC × 35c	PFS, OS	ORR, Safety, QoL
Makker et al., 2023 [21]	309–KEYNOTE-775, NCT03517449 (secondary analysis)	N = 827 Lenvatinib + Pembrolizumab: 411/827 dMMR: 130/827	Advanced, recurrent, or metastatic endometrial cancer (except carcinosarcoma and sarcoma)	Lenvatinib 20 mg once daily + Pembrolizumab 200 mg q3w or TPC × 35c	PFS, OS	ORR, Safety, QoL
Yonemori et al., 2022 [22]	309–KEYNOTE-775, NCT03517449 (Japanese subset of patients)	N = 104 Lenvatinib + Pembrolizumab: 52/104 dMMR: 8/104	Advanced, recurrent, or metastatic endometrial cancer (except carcinosarcoma and sarcoma)	Lenvatinib 20 mg once daily + Pembrolizumab 200 mg q3w or TPC × 35c	PFS, OS	ORR, Safety

DC, disease control; dMMR, deficiency mismatch repair; EC, endometrial cancer; EP, endpoint; mo, months; ORR, Objective Response Rate; OS, overall survival; PBC, platinum-based chemotherapy; PFS, progression-free survival; QoL, quality of life; RD, response duration; TPC, treatment of physician’s choice; TSPD, time to second progressive disease.

**Table 2:** Results of trials.

Study Name	PFS	OS	Discontinuation Rate	Dose Reduction	Temporary Interruption
RUBY [17]	dMMR: 61.4% (24 mo) [95% CI 46.3–73.4 mo] OP: 36.1% [95% CI, 29.3–42.9 mo]	dMMR: 83.3% (24 mo) [95% CI 66.8–92.0 mo] OP: 71.3% (24 mo) [95% CI, 64.5–77.1 mo]	17.4%	NA	NA
NRG-GY018 [18]	dMMR: 74% (12 mo) pMMR: 13.1 mo (median)	NA	57%	NA	NA
DUO-E/ENGOT-EN10 trial [19]	Durvalumab: 48.5% (12 mo) Durvalumab + Olaparib: 61.5% (12 mo) dMMR: - Durvalumab 67.9% (12 mo) - Durvalumab + Olaparib: 70% (12 mo)	Durvalumab: 84.2% (12 mo) Durvalumab + Olaparib: 87.7% (12 mo)	Durvalumab: 20% Durvalumab + Olaparib: 24.4%	Durvalumab: 6% Durvalumab + Olaparib: 27.3%	Durvalumab: 54.5% Durvalumab + Olaparib: 68.9%
309–KEYNOTE-775 [20]	pMMR: 6.6 mo [95% CI, 5.6–7.4 mo] (median) OP: 7.2 mo [95% CI, 5.7–7.6 mo] (median)	pMMR: 17.4 mo [95% CI, 14.2–19.9 mo] (median) OP: 18.3 mo [95% CI, 15.2–20.5 mo] (median)	33%	66.5%	69.2%
309–KEYNOTE-775 (secondary analysis) [21]	pMMR: 6.7 mo [95% CI, 5.6–7.4 mo] (median) OP: 7.3 mo [95% CI, 5.7–7.6 mo] (median)	pMMR: 18.0 mo [95% CI, 14.9–20.5 mo] (median) OP: 18.7 mo [95% CI, 15.6–21.3 mo] (median)	39.2%	72.2%	71.9%
309–KEYNOTE-775, NCT03517449 (Japanese subset of patients) [22]	pMMR: 5.6 mo [95% CI, 3.7–7.6 mo] (median) OP: 7.2 mo [95% CI, 3.7–8.8 mo] (median) dMMR: 71.4% (6 mo)	pMMR: 16.7 mo [95% CI, 11.8-NR mo] (median) OP: NR [95% CI, 12.1-NR mo] (median) 87.5% (12 mo)	36.5%	82.7%	63.5%

dMMR, deficiency mismatch repair; mo, months; NA, not available; NR, not reached; OP, overall population; OS, overall survival; PFS, progression-free survival; pMMR, proficiency mismatch repair.

**Table 3:** TRAEs (treatment-related adverse effects).

Study Name	Any Grade AE	Most Common AE	AE Leads to Discontinuation	Grade ≥ 3 AE	AE Causing Death
RUBY trial [17]	100%	- Nausea 53.9% - Alopecia 53.5% - Fatigue 51.9%	- Maculo-papular rush 1.2% - Infusion-related reaction 1.2%	70.8%	2.1%
NRG-GY018 [18]	dMMR: 98.2% pMMR: 93.5%	- Fatigue: 71.6% (dMMR), 63.4% (pMMR) - Peripheral sentorial neuropathy: 65.1% (dMMR), 55.4% (pMMR) - Anemia: 57.8% (dMMR), 55.1% (pMMR)	NA	dMMR: 63.3% pMMR: 55.1%	dMMR: 0.9% pMMR: 2.2%
DUO-E/ENGOT-EN10 trial [19]	Durvalumab: 98.7% Durvalumab + Olaparib: 99.6%	- Durvalumab arm: anaemia 47.7%, nausea 40.9%, fatigue 43% - Durvalumab + Olaparib arm: anaemia 61.8%, nausea 54.6%, fatigue 54.2%	NA	Durvalumab: 54.9% Durvalumab + Olaparib: 67.2%	NA
309–KEYNOTE-775 [20]	99.8%	- Hypertension 64.0% - Hypothyroidism 57.4% - Diarrhoea 54.2%	- Hypertension 2% - Asthenia 1.7%	88.9%	5.7%
309–KEYNOTE-775 (secondary analysis) [21]	99.8%	- Hypertension 65.0% - Hypothyroidism 58.9% - Diarrhoea 55.7%	- Hypertension 2% - Diarrhoea 2%	90.1%	6.4%
309–KEYNOTE-775, NCT03517449 (Japanese subset of patients) [22]	100%	- Hypertension 78.8% - Hypothyroidism 75.0% - Proteinuria 63.5%	NA	90.4%	0%

AE, adverse effect; dMMR, deficiency mismatch repair; NA, not available; pMMR, proficiency mismatch repair.
